# Advancing global chemical education through interactive teaching tools

**DOI:** 10.1039/d2sc01881k

**Published:** 2022-05-11

**Authors:** Francesca M. Ippoliti, Jason V. Chari, Neil K. Garg

**Affiliations:** Department of Chemistry and Biochemistry, University of California Los Angeles California 90095 USA neilgarg@chem.ucla.edu

## Abstract

This perspective highlights our recent efforts to develop interactive resources in chemical education for worldwide usage. First, we highlight online tutorials that connect organic chemistry to medicine and popular culture, along with game-like resources for active learning. Next, we describe efforts to aid students in learning to visualize chemical structures in three dimensions. Finally, we present recent approaches toward engaging children and the general population through organic chemistry coloring and activity books. Collectively, these tools have benefited hundreds of thousands of users worldwide. We hope this perspective promotes a spirit of innovation in chemical education and spurs the development of additional free, interactive, and widely accessible chemical education resources.

## Abstract 2

This perspective highlights the development of interactive chemical education resources for worldwide usage. We hope to promote a spirit of innovation in chemical education and spur the development of new chemical education resources.

## Graphical abstract

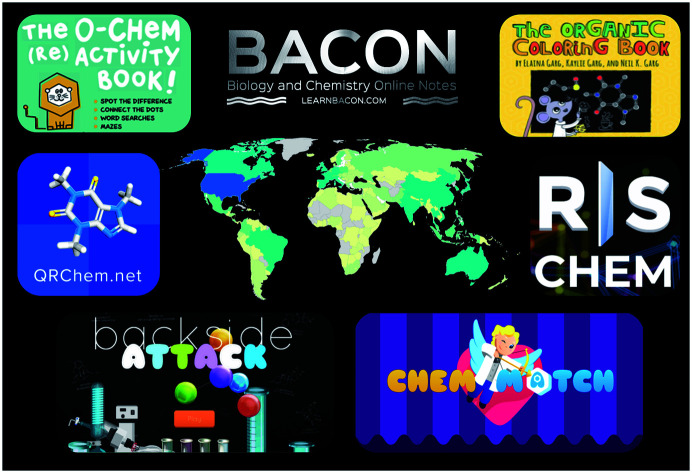

## Introduction

As researchers, we devote the majority of our professional efforts, and often even free time, to thinking about and addressing challenges we face in the laboratory. Simultaneously, we have come to realize the impact we researchers are poised to make—and have an obligation to make—when it comes to science education and scientific literacy. Scientific literacy, as described by the National Academy of Sciences, “means that a person can ask, find, or determine answers to questions derived from curiosity about everyday experiences. It means that a person has the ability to describe, explain, and predict natural phenomena.”^1^ In other words, scientific literacy pertains to more than simply the ability to recite scientific facts; it extends to an individual's own judgment and decisions when it comes to science and therefore represents a complex, but critical issue. Heightening the value and importance of scientific literacy, scientific misinformation represents a growing problem in the age of social media.^2^

The key to addressing global scientific literacy lies in how we educate and, importantly, ensuring the accessibility of educational resources. As we consider chemical education, an all-too-common historical approach involves memorization and recitation of facts. This approach can be counterproductive in many ways and lead to negative perceptions by students. With regard to accessibility, resources for chemical education vary widely throughout the world, with many students not having access to costly textbooks, molecular model kits, or sometimes instructors.

Toward addressing these challenges, we have taken a keen interest in developing non-traditional educational platforms^3^ that focus on growing students' critical thinking and problem-solving skills. In particular, these tools serve to put students “in the driver's seat” by compelling them to actively engage and connect with the material. Furthermore, many of our resources seek to make chemistry relatable and engaging to the students by incorporating real-world applications and examples. Engagement is known to correlate well with learning outcomes.^4^ In addition, we have sought to create educational tools that are available online for worldwide use given that 4.95+ billion people have internet access already (and this figure is expected to grow).^5^ Moreover, the COVID-19 era of virtual education has created an even greater need for readily accessible online teaching materials. Indeed, studies have demonstrated a marked decline in student engagement in virtual settings during the pandemic.^6^ The creation of effective online teaching materials not only benefits students studying chemistry, but also engages the broader population in our field.

In this perspective, we highlight our efforts to develop interactive and widely accessible resources that are available to all students. These resources were each created by teams comprised of individuals with diverse sets of experiences and backgrounds. This included undergraduate and graduate students, postdoctoral researchers, high school students, and even children. This allowed us to target a variety of audiences by better understanding challenges that exist in different stages of learning scientific topics. Critical to all of these projects was our collaboration with Dr Daniel Caspi of Element26, Inc. who is a computing expert and master of creating user interfaces across various platforms.

## Development and application of interactive online learning tools

An important means of engaging students in chemistry is in relating course content to students' everyday lives. As an example, connecting organic chemistry to biology and popular culture helps to demonstrate that the course material is highly relevant to the students' lives, even if they do not intend to pursue further studies in that field. In particular, we hoped to engage students whose studies are not primarily focused in the area of chemistry.

**Fig. 1:**
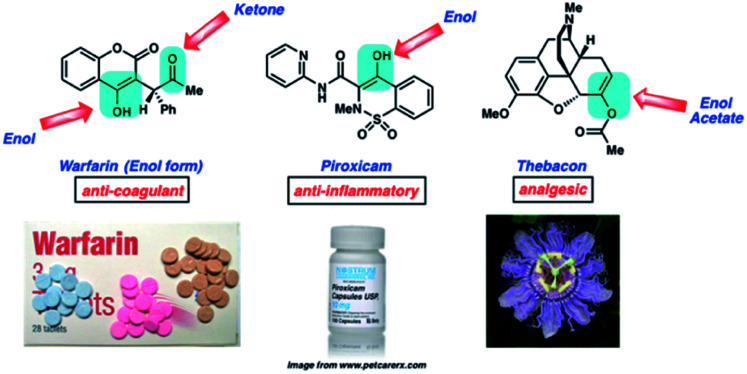
A selected example from BACON (Biology and Chemistry Online Notes), an online set of tutorials that connect organic chemistry to human health and popular culture.

With this in mind, we sought to create an online platform that connects chemistry concepts to medicine and popular culture. This ultimately led us to create BACON (Biology And Chemistry Online Notes, https://learnbacon.com), an online set of tutorials that serve as a vehicle for students to make extensive connections between organic chemistry, human health, and popular culture (Fig. 1).^7^ BACON consists of sixteen learning modules that cover organic chemistry topics such as ‘Stereochemistry and Chirality,’ ‘Diels–Alder and Pericyclic Reactions,’ and ‘Polymers.’ Each module highlights several examples of the topic in both medicine and popular culture to deepen students' understanding of both the concepts and their importance. We also strive to keep these modules relevant to current topics, such as highlighting CRISPR gene editing technologies—which were the subject of the 2020 Nobel Prize in Chemistry^8^—as well as updated popular culture references that mention these scientific breakthroughs. Importantly, the tutorials also highlight members of the scientific community from underrepresented backgrounds in chemistry.

As mentioned earlier, we have prioritized the creation of online teaching tools for chemical education in order to not only impact our local community, but also countries around the world. With respect to its global impact, BACON has been used by over 228 000 people in 169 different countries,^9^ and over 165 universities have integrated BACON into some of their chemistry curricula.^10^

**Fig. 2:**
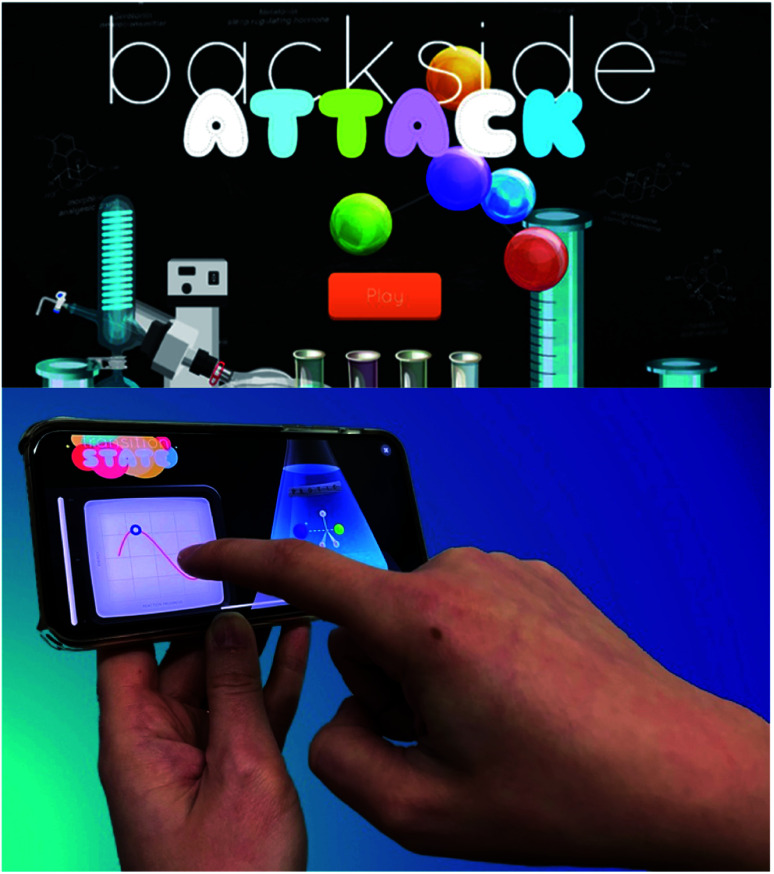
Backside Attack, a smartphone game that challenges students to master the S_N_2 reaction, an important reaction in undergraduate organic chemistry coursework.

In addition to online learning modules, we have also sought to develop game-based learning tools that create fun and interactive learning environments for students. For example, the smartphone app Backside Attack (Fig. 2) was launched to help students interact directly with a fundamental chemical reaction, the S_N_2 reaction. This topic was specifically chosen as it involves several critical concepts that appear throughout undergraduate organic chemistry curricula. These concepts include nucleophilicity, electrophilicity, and the effect of sterics and solvents on reaction outcomes. This free smartphone app was conceived of and developed by undergraduate students who had used BACON in their organic chemistry coursework. These students appreciated the connections between biology, popular culture, and chemistry, but also envisioned a game-like resource that could help students learn the nuances of a new concept through an entertaining and interactive format. Backside Attack involves an enticing user interface, where participants launch a nucleophile from a syringe into a “solution” containing an electrophile with which it can react. The user is then prompted to draw an arrow-pushing mechanism for the reaction. Next, the user must physically tap repeatedly on the screen to simulate the energy required to overcome the activation barrier for the S_N_2 reaction. Finally, the user is tasked with answering a textbook-style problem about the material covered in the level. This free application allows students to explore each aspect of the S_N_2 reaction, which is anticipated to provide greater engagement with and absorption of the material. The “Chem Yourself” feature provides an opportunity for users to share their progress with their friends by creating a fun and personalized, chemistry-themed image. Other innovative games have also been created to help students connect with chemistry concepts, such as those developed by Alchemie.^11^ Overall, these tools provide exciting opportunities for students to learn challenging concepts in an interactive and fun environment.

## Teaching in three dimensions

A crucial skill for any chemistry student is the ability to visualize chemical structures. In particular, understanding chemical structures in two dimensions and carrying this knowledge into three dimensions is central to introductory coursework in chemistry. This involves concepts such as Valence Shell Electron Pair Repulsion (VSEPR) theory, chirality, and stereochemistry. Although these concepts make up a critical foundation of chemistry education, they consistently represent major obstacles for students, with reports of these challenges dating back to the 1940s.^12^ Common teaching tools such as physical model kits, while powerful in many cases, can sometimes be inconvenient or costly. Alternatively, one might consider leveraging modern technology to address this challenge. This offers an immense opportunity for chemistry educators to innovate by developing new interactive tools that facilitate the visualization of chemical structures. Such tools offer the potential to not only aid in students' understanding of three-dimensional structures, but also in generating excitement about these fundamental concepts.

**Fig. 3:**
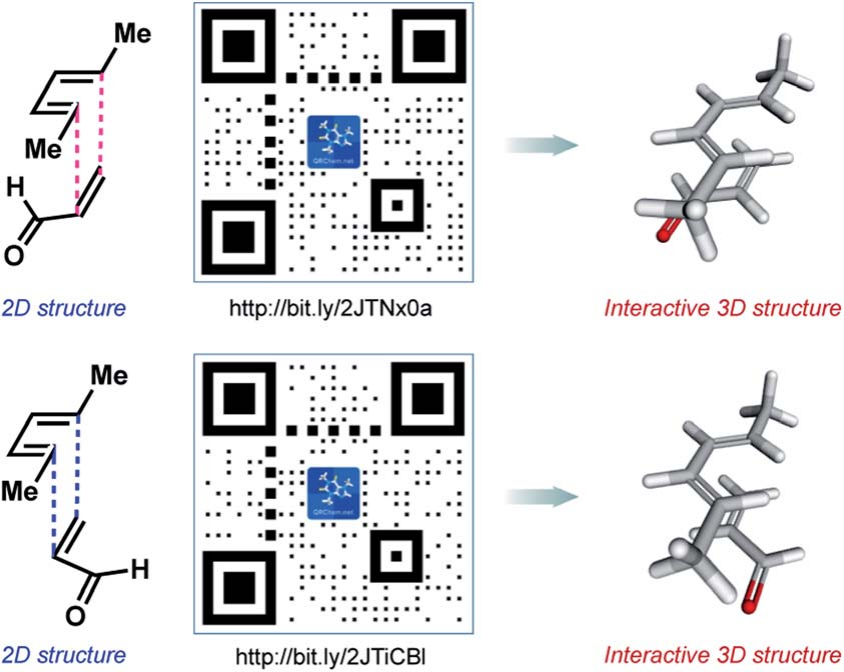
QR Chem, a site that allows students, instructors, and researchers to create QR codes that link to interactive 3D structures. In this example, two potential scenarios for the classic Diels–Alder reaction are depicted, each leading to different isomeric outcomes.

Given the need for alternative approaches to teach students how to visualize chemical structures, we sought to develop a resource that takes advantage of smartphone technology. Our vision was to create a resource that would allow students to visualize any chemical structure instantly and without the need for a physical model kit. Toward achieving this, we opted to leverage the wide utility of quick-response (QR) code technology and in 2018, launched QR Chem (https://QRChem.net).^13^ This project was initially led by a team of undergraduate students, who were later joined by graduate students for further development of the content. This site allows educators and researchers to create QR codes, as well as bit.ly URLs, that link directly to a three-dimensional structure of interest (Fig. 3). As an example, an instructor may create and present a QR code to a class of students, each of whom can then scan the QR code using their smartphone's built-in camera to open the interactive structure directly on their device. The student is then able to rotate, as well as zoom-in and zoom-out on, the chemical structure in order to compare it to a two-dimensional representation that is also displayed on the screen (an example of this is shown in Fig. 3).

Since the initial launch of QR Chem, we have created a ‘Molecule of the Day’ page for the site that showcases over fifty different molecules with important societal impact. This module takes the form of slide-based presentations with embedded QR codes that link to three-dimensional structures, as well as provide interesting information about each molecule. Additionally, the ‘Lesson Plans’ page on the site contains useful slide-based presentations with embedded QR codes that cover a variety of topics that rely on the visualization of chemical structures (*e.g.*, the Diels–Alder reaction, as depicted in Fig. 3). It should also be noted that any user can generate a sharable QR code by simply uploading a 2D and 3D structure file, or submitting a valid PubChem CID number for virtually any molecule of interest. In our own experience, QR Chem helped to facilitate interactive learning in courses taught remotely.^14^ QR codes generated from QR Chem can also be found in Wikipedia entries, as well as in some textbooks.^15^ Excitingly, QR Chem has been used by over 62 000 people in over 150 countries.

**Fig. 4:**
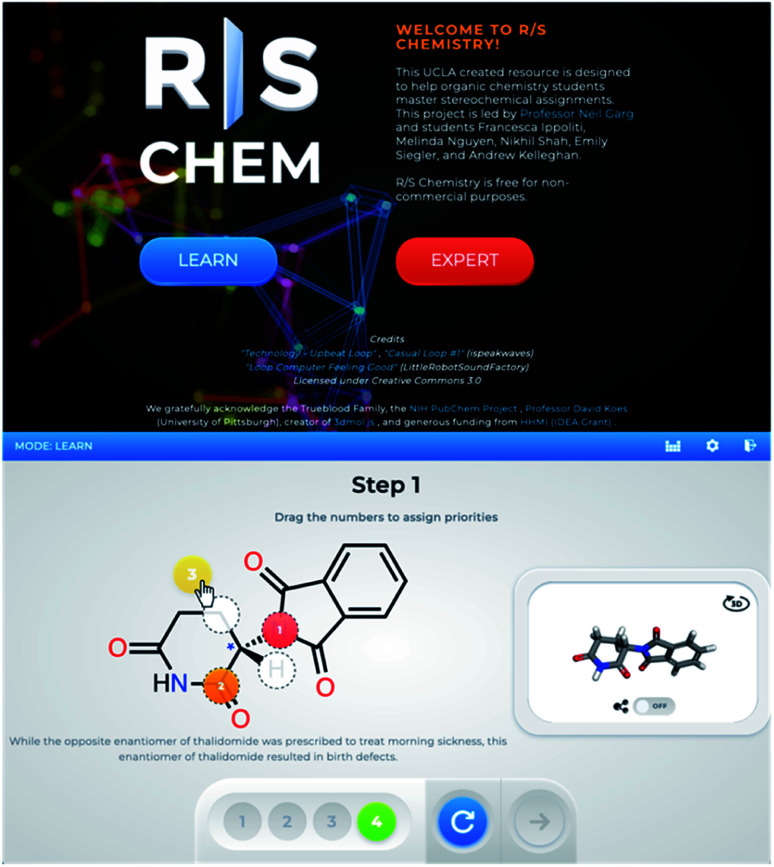
R/S Chemistry, a resource for students to practice assigning stereocenters in a game-like environment.

In addition to being able to visualize chemical structures, assigning a stereocenter on a molecule is a critical skill for organic chemistry students. We sought to create a learning tool to help teach this skill while also being more interactive and engaging than textbook-based approaches. We envisioned that a game-like interface could serve to achieve this, as such a platform would provide the ability for students to receive direct feedback as they learn how to assign stereocenters and put their knowledge to the test. R/S Chemistry (https://RSChemistry.com), launched in 2019, represents our interactive solution to this challenge of learning how to assign stereocenters (Fig. 4). As with QR Chem, the idea for this resource was originally devised by undergraduate students, with graduate students joining the team later to develop the content. It features multiple levels of gameplay with both a guided ‘Learn’ mode and a timed ‘Expert’ mode for all levels of student mastery. R/S Chemistry leverages the visualization technology used in QR Chem to include interactive three-dimensional structures that assist in the stereochemical assignments. In selecting example molecules for the game, we sought to highlight compounds with broad societal impact, such as medicines and fragrances, to help students connect with the content. Of note, such connections are often missing in traditional teaching tools, further underscoring the need for alternative educational approaches that showcase them. Since its launch, R/S Chemistry has been used by more than 21 000 people in over 100 countries.^16^

Advances in modern technology continue to provide new opportunities for chemical visualization, and, in turn, new areas for innovation. Virtual Reality (VR) and Augmented Reality (AR) technology enable the creation of immersive environments for teaching in three dimensions. Excitingly, these technologies offer the ability to teach abstract concepts that may be very challenging to describe in detail using traditional teaching tools. Several examples of immersive VR^17,18^ and AR^19^ technology in chemical education have been reported, many of which provide compelling evidence that implementation of virtual reality tools improve student performance. For example, Kurushkin and co-workers have demonstrated that the implementation of a virtual reality program called MEL Chemistry VR in coursework at ITMO University in Russia has improved undergraduate students' ability to grasp challenging concepts pertaining to atomic structure.^12*a*^ Additionally, Diaconescu and co-workers developed a VR laboratory session to teach advanced inorganic chemistry students about metal coordination and molecular orbitals, which resulted in higher exam scores by the students who participated in the VR session compared to those who did not.^12*b*^ Compelling smartphone-based applications that employ AR technology have also been developed, including Isomers AR by Alchemie,^20^ a game that allows users to build and discover new molecules in 3D without the need for a specialized headset. We have also begun to adapt content from QR Chem into a virtual reality interface (https://VRChem.net) to create an immersive environment where students can view and interact with three-dimensional structures, as well as learn about important molecules that connect to their everyday lives.

## Reaching new audiences

**Fig. 5:**
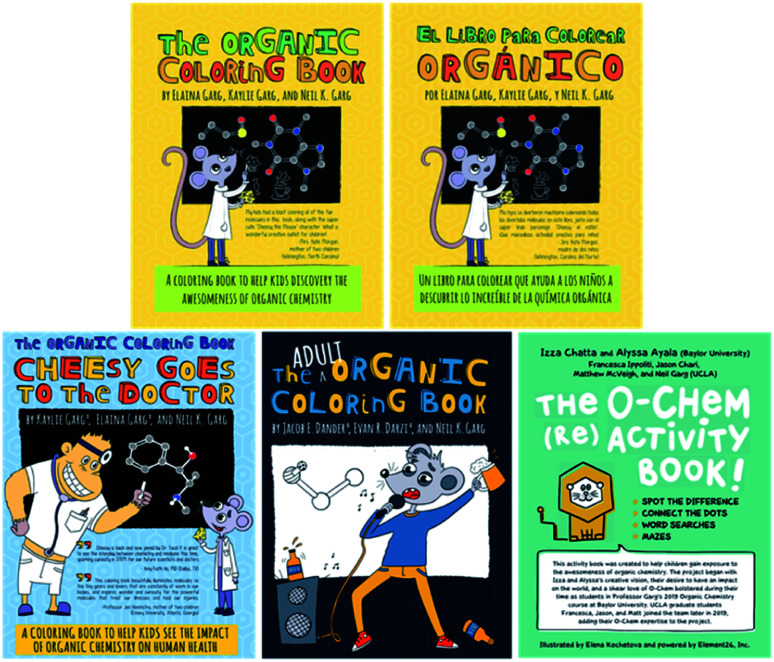
The Organic Coloring Book series and The O-Chem (Re)Activity Book are designed to connect organic chemistry to the everyday lives of both children and adults.

A crucial aspect of addressing scientific literacy lies in connecting with audiences outside of higher education. As a personal anecdote, one of us (N. K. G.) noticed that his daughter was particularly afraid of chemicals from an early age. Before trying a new food, she would often ask, “does it have chemicals in it?” and express wariness about the food. However, once it was explained to her that chemicals make up everything around her, including her favorite things, she became more curious about which chemicals were part of her daily life. We saw this as an opportunity to expand our impact as scientists and educators to a younger generation. In particular, we envisioned that introducing chemistry to children through fun and engaging activities could serve to reduce the negative association they have with the word “chemical.” In considering interactive activities that children are familiar with, we opted to develop a coloring book about organic molecules. The final product, The Organic Coloring Book, features molecules such as sucrose, cellulose, and chlorophyll (Fig. 5). Exposing children to the connection between science and everyday life serves to increase children's curiosity about the world around them and hopefully spark lifelong interest in science. We have also received positive feedback from parents, who note that the book also helped them learn about chemistry in the world around them. The Organic Coloring Book is accessible for purchase online,^21^ although we regularly distribute free copies throughout the world. To make this resource accessible to a broader global population, we have also published a Spanish edition of The Organic Coloring Book, entitled El Libro Para Colorear Orgánico.^22^

The success of The Organic Coloring Book, as well as its medicine-themed sequel (Cheesy Goes to the Doctor),^23^ led us to wonder whether we could develop similar resources to reach adults as well. Indeed, we felt that our motivations for creating the organic coloring book for children could also be extended to adults. As an example, “chemical-free” products are often marketed as desirable despite the impossibility of a product that does not contain chemicals. We ultimately created The Adult Organic Coloring Book,^24^ highlighting molecules that have relevance to adult life, such as ethanol and creatine, an amino acid used for muscle growth.

Another interactive resource that we felt would be exciting to children is an activity book, which we envisioned would consist of chemistry-themed exercises. Similar to The Organic Coloring Book, each of these activities would serve to expose children to chemistry and its connection to daily life. We ultimately developed The O-Chem (Re)Activity Book, which is comprised of fun exercises including connect-the-dots, word searches, mazes, and ‘spot the difference,’ each of which highlight chemistry in the world around us. The book is available free of charge online as a downloadable PDF,^25^ allowing parents around the world to download the book and enjoy it with their children.

**Fig. 6:**
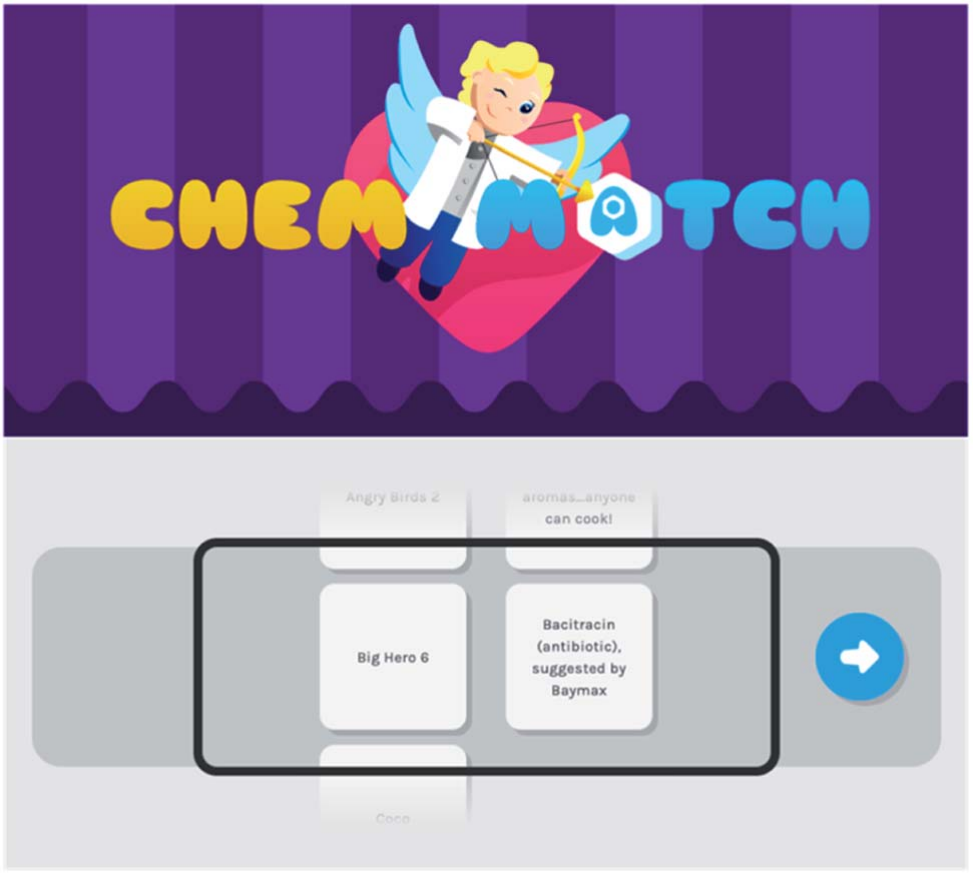
ChemMatch, an online matching game that serves as an educational resource for children, students, and adults.

Additionally, we recently launched ChemMatch (https://ChemMatch.net), an online matching game that includes several chemistry-oriented subjects designed to reach various audiences (Fig. 6). This includes topics relevant to all audiences such as “Chemistry in Your Life” and “Kids Movies,” as well as topics for high school chemistry students and college general chemistry students including “Elements” and “Polyatomic Ion Charges.” Organic chemistry-specific categories are also included.

In addition to the books and online resources created by our lab, there have been a multitude of chemistry books and activities that connect children with the science found around them.^26^ Introducing chemistry to children at a young age can have a lasting impact on the importance of science in their worldview. More broadly, developing resources that reach a wide audience can also help to promote a positive public perception of chemistry among non-scientist adults. We look forward to seeing future efforts in this area.

## Global impact and innovation in education

**Fig. 7:**
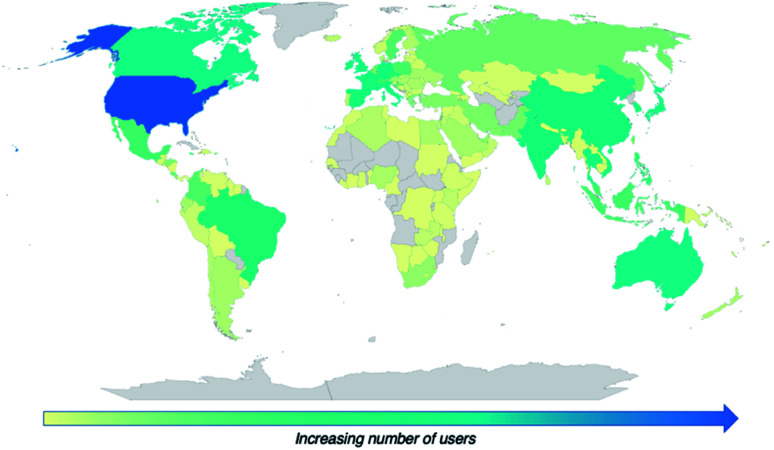
Map of combined users of QR Chem, R/S Chemistry, and ChemMatch worldwide (data from Google Analytics).

A crucial objective of our efforts in education is reaching audiences across the globe. Accordingly, we have evaluated the usage of our web-based resources in countries worldwide and have been gratified to observe that our online resources have been used all around the world. To illustrate this, Fig. 7 depicts global usage of QR Chem, R/S Chemistry, and ChemMatch, which have a combined total of over 90 000 users in 157 countries. These widely accessible resources not only engage chemistry students, but also present a step toward educating the more general population. We hope to continue expanding the reach of these resources around the world.

## Conclusions

We have developed a multitude of non-traditional chemical education resources, including a smartphone application, several websites, and children's books. These tools transcend classic teaching methods, such as those primarily focused on textbooks and memorization. Instead, we largely rely on internet-based technologies that enable critical thinking and engagement in order to help students learn about and appreciate chemistry. In addition, our educational resources for children and the general population contribute to the widespread societal challenge of improving scientific literacy. Collectively, the resources we have developed so far have positively benefitted hundreds of thousands of people all over the world.

Our goal in writing this perspective is not to simply advertise our educational materials. Instead, we offer our view as active researchers of the potential impact we, as a community of scientists, can make as we think about science education on a global scale. It is incumbent upon all of us to contribute to science education worldwide. Such efforts not only benefit our own fields by engaging individuals in chemistry, but more importantly, have the capacity to benefit society in the long-term. We should also prioritize the development of resources that are fun, engaging, promote critical thinking skills, and are accessible to everyone.

Lastly, we offer sentiments regarding “innovation,” a term frequently employed in academia. Although there exists a strong spirit of innovation in scientific research, a comparable sentiment is notably lacking in many research-intensive colleges and universities when discussing chemical education. By choosing to prioritize innovation in chemical education, we stand to improve the student experience, educate the scientific leaders of the future, enhance global education and scientific literacy, and even strengthen the public perception of our fields.

## Related links

**BACON:** https://learnbacon.com/

**Backside Attack:** https://apps.apple.com/us/app/backside-attack/id1278956096/

**QR Chem:** https://qrchem.net/

**R/S Chemistry:** https://rschemistry.com/

**ChemMatch:** https://chemmatch.net/

**Organic coloring book series:** https://www.amazon.com/dp/B08NXL4SWP/

**The O-Chem (Re) Activity Book:** https://garg.chem.ucla.edu/ochem-re-activity/

**Element26:** https://www.element26.net/

## Author contributions

All authors were involved in the preparation of the manuscript. The final manuscript was approved by all authors.

## Conflicts of interest

There are no significant conflicts to declare.

## Supplementary Material
